# *‘Things we are expected to just do and deal with’*: Using the medical humanities to encourage reflection on vulnerability and nurture clinical skills, collegiality, compassion, and self-care

**DOI:** 10.1007/s40037-022-00724-w

**Published:** 2022-08-12

**Authors:** Michaela Kelly, Johanna Lynch, Penny Mainstone, Alison Green, Nancy Sturman

**Affiliations:** General Practice Clinical Unit, The University of Queensland, Brisbane, Australia

**Keywords:** Reflective practice, Creative enquiry, Doctor-patient relationship, Medical student and doctor’s health

## Abstract

The *Vulnerability in Medicine *(ViM) program was developed to provide protected time and psychologically safe spaces for third-year medical students to consider challenges in the doctor-patient relationship and the clinical workplace. A suite of discussion-prompts presented in a small-group learning environment provides a springboard for students to reflect on their development as clinicians, understand the personhood of their patients, explore the therapeutic relationship, and consider emotional responses and personal, cultural, and social assumptions that impact on care. The program supports students to recognise vulnerability in themselves, the patient, their tutors, and the wider clinical team, as they face the challenge of aligning the clinician they want to become with ideals of professionalism and the imperfect clinical workplace. This 6‑week program focuses on the vulnerability of patients, students, and doctors in a weekly tutorial interposed with clinical placements primarily in geriatric, rehabilitation, or palliative medicine. The tutorials draw from the medical humanities and use experiential, reflective, and narrative learning techniques. They are facilitated by generalist clinicians who model their own vulnerability, humanity, and reflective practice by sharing tutorial tasks equally with students. Students report feeling supported, and appreciate the opportunity to discuss ethical, psychosocial, and emotional aspects of medicine whilst reflecting on what medical practice means to them. Tutors experience a deeper appreciation of student journeys and their own vocations as clinicians and teachers. The sharing of vulnerability exposes the humanity of patients, students, and clinicians, and sustains our whole-person approach to the care of patients, students, and ourselves.

## Background

To flourish as future professionals, medical students need protected time and safe spaces to reflect on, and discuss, their experiences [1–3]. Swept into the vortex of crowded curricula and high-stakes assessments, they have little time to contemplate themselves, their patients, doctor-patient relationships, or the distressing, moving, or even transcendent experiences of the imperfect clinical workplace [1–3].

Several programs have been developed to provide such spaces, tailored to local needs and available resources. These include: Schwartz Rounds for Medical Students [4], focusing on social and emotional aspects of healthcare; the Healer’s Art program [5, 6], focusing on values, experiences, meaning, collegial support, and healing relationships; and Mindful Medical Practice[7] focusing on awareness of self, context, and the patient as a whole person. Such programs foster transformative learning though emotional engagement, embracing multiple viewpoints, and supporting reflective practice and professional identity formation[8]. Most engage with the medical humanities and promote student self-care strategies to reduce rates of anxiety, depression, and burnout in medical professionals and students[9].

We describe below our own approach, which also draws inspiration from whole-person care [10, 11], reflective practice [13], Balint groups [12, 14–16] compassion-cultivation training [17], and narrative medicine [18, 19].

Since 2019, six experienced generalist clinician-tutors have facilitated a weekly tutorial program to 328 students undertaking 6‑week clinical placements in geriatric medicine, palliative care, rehabilitation medicine, or refugee health. These *Vulnerability in Medicine* (ViM) tutorials are carefully nurtured safe spaces for students to venture courageously outside the biomedical, into the humanities and creative arts. We aim to deepen student understanding of patient experiences and the doctor-patient dynamic, as they engage in reflective practice. Novel aspects of our program include its inclusion as a course requirement, its weekly schedule throughout a clinical placement, the allocation of tutorial tasks to both students and tutors creating a culture of shared leadership, the deliberate flattening of the teacher-student power differential, the focus on ethical dilemmas in the clinical learning environment, and the use of creative enquiry and expression [20].

## Program design

The 2‑hour tutorials are a compulsory course component. Students are encouraged to debrief about challenges or concerns from the clinical environment and are rostered to one or more tasks each week (see Tab. 1). All tasks have been designed and refined iteratively through student and tutor feedback and each is modelled by the tutor in the first tutorial. The first task is choosing an opening reflection or meditation. Other tasks include leading discussions on a ‘question-of-the-week’, an excerpt of medical literature, and an ethical dilemma collated by the course coordinator. The only graded tutorial task is the *Understanding the Person Discussion*, in which students, following the example of their tutors, present an in-depth ‘whole-person’ interview with a patient about their childhood, family, working life, important life events, experiences of healthcare, the impact of illness on their life, thoughts about their future, and what brings them meaning. Students share their personal response to the patient’s narrative, while encouraging curious questions from the group about the person and their therapeutic relationships. The final tutorial task is the sharing of a piece from the humanities or creative arts chosen for its personally meaningful connection to the theme of vulnerability. During the 6 weeks, students and tutors are encouraged to practice self-care, to reflect on their lived experience as students and clinicians, and to re-engage with enriching or restorative creative activities, hobbies, or interests they may have set aside due to study or work. In the final tutorial, additional time is provided for everyone to share a personal creative contribution.

**Table 1:** Structure of the *Vulnerability in Medicine* tutorial program

Program duration	6 weeks to align with the length of the student’s clinical placement
Tutorial frequency	Weekly
Tutorial duration	2‑hour tutorial, final tutorial 2 ½ hrs
Students	Third-year students in a graduate medical program undertaking clinical placements in geriatric medicine, palliative care, rehabilitation medicine, and refugee health
Tutors	Generalist clinicians working in different clinical workplaces to the students’ clinical placements and not directly involved in students’ clinical assessment. This assists in minimising power-differentials and fostering an open tutorial environment
Number of students	6–9 students per tutorial group, one tutor per group
First tutorial	Includes introductions and formulation of group rules. The tutor leads all tasks in the first tutorial. This helps students understand the tutor’s expectations for the task and allows tutors to bring their patients, challenges, and reflections into the tutorial space which helps establish safety and models vulnerability and openness
Check-in, debrief	Each tutorial provides protected time for students and tutors to bring interesting observations, challenges, or concerns from their week of clinical placement or work for sharing in the tutorial space
Rostered tasks^a^	Description
Tutorial opening	Tutorials open with a general reflection of the previous week or if the group desires, a guided mindful meditation chosen by the person rostered to lead the task (max. 5 min )
Lead for question of the week discussion	Each week a question is provided for discussion. For example: *What forms of vulnerability do you find difficult to be near? How can you care for yourself when you are near that form of vulnerability*?
Lead for book reading	Published reflections written by doctors or excerpts from such reflections, e.g. ‘Perspectives’ from the New England Journal of Medicine, *Kitchen Table Wisdom* by Rachel Remen, excerpts from a range of novels written by doctors
Lead for ethical scenario discussion	Short ethical scenarios are provided focusing on professional boundaries, microaggressions and threats to professionalism
Lead for understanding the person discussion	Students summarise and reflect on their experience of conducting an in-depth interview with a patient in preparation for the tutorial. In this interview students aim to understand the person through exploring current circumstances, important life events, values, beliefs, what provides meaning, healthcare experiences, impact of illness on the patient and loved ones, and social and structural determinants of health. The student usually needs to meet with the patient several times and is careful to only discuss content the patient is comfortable discussing. Permission is sought from the patient to share their story within the tutorial group
Tutorial closure	The tutorial closes with the rostered member of the group sharing an image or text from the humanities or creative arts that is meaningful to them or connects with their experiences in the previous week
Creative-reflective contribution	During the 6 weeks of the program, group members (including the tutor) draw on their reflective, analytical, or creative skills and talents (particularly those they may have neglected due to their studies or work) to develop an individual reflective contribution. A wide range of media can be used, for example, poetry, short story, visual arts (drawing, painting, sculpture, craftwork, photograph/montage, collage etc.), creative short film, music composition/performance, game design and dramatic performance or experiences of cooking, physical pursuits, or gaming. In the final tutorial this creative work is shared with the group. The sharing must not exceed 5 min but must include an explanation of how the work articulates and reflects what vulnerability means to them

^a^ Please note—the question of the week, book reading excerpt and ethical scenarios are provided to students, collated by the course coordinator, and informed by suggestions from tutors and students. Tutorial participants are rostered to lead the discussion of specific tasks

## Impact on student learning and experience

Ethics approval was obtained for an evaluation of this program; the project complies with the provisions contained in the *National Statement on Ethical Conduct in Human Research* and with the regulations governing experimentation on humans (The University of Queensland Institutional Human Research Ethics Approval Number: 2019000169). Most students surveyed indicated overall satisfaction with the program (86% of students were very satisfied/satisfied, 10% neutral and 4% dissatisfied/very dissatisfied, response rate 169/190, 88.9%). Free text responses indicated four themes: a road less travelled; openness and candour; self-care and being cared for; and connection and collegiality.

### The road less travelled

Students acknowledged the cognitive shift from a biomedical focus to a ‘whole-person’ approach embracing the psychosocial domain. A small number of students reported preferring a biomedical emphasis, in contrast to most students who welcomed the shift:

I like that it is a breath of fresh air discussing the biopsychosocial model of medicine and not just the medical part. I enjoy the ethical discussions, the wide variety of ideas from my peers, and I enjoy that it is a non-judgemental space (2019, student 54).

### Openness and candour

Many students appreciated the safe and non-judgemental space to acknowledge challenges and powerful emotions in the face of patient suffering, and the support to respond compassionately and curiously. Some students reported initially struggling to share their thoughts and feelings but developing confidence as the program continued. Students witnessed patient struggles and challenges, with some students observing their first patient death. Most listened to narratives of trauma, loss, and sadness, using tutorials to explore or ‘sort through’ their feelings. Discussion topics also included moral distress, and concerns about workplace culture.

I loved that it gave us an outlet to talk about our vulnerabilities, stressors, and fears in a safe space. These are all the things we’re expected to just do and deal with as a doctor, but they are never talked about (2020, student 38).

### Self-care and being cared for

Students expressed feelings of being cared for personally by clinicians and peers who were interested in their well-being. Students appreciated the relaxed discussions with peers and a clinician external to their clinical placement, away from the busy clinical environment. They welcomed opportunities to discuss medical student and doctor health and explore self-care strategies.

The environment … allowed time for us to mentally sort through the emotionally overwhelming experiences and derive protective mental health coping strategies (2020, student 41).

### Connection and collegiality

Students enjoyed learning about their tutor’s experiences with patients, including their challenges and vulnerabilities. Tutor modelling of tutorial tasks made them seem more equal participants in the learning space.

Felt like I was on equal ground and being treated as such … encouraged everyone to be more open about their own experiences in a comfortable way (2019, student 8).

By having tutors have an active role it was helpful in comparing my level of reflection and effort to what can be potentially reached (2019, student 15).

Students appreciated learning that other students were experiencing similar challenges and reported a deeper and more meaningful sense of collegiality.

Being able to hear and discuss with other students about their experiences … helped me to reflect more deeply on my own … helped me feel less alone (2020, student 13).

## Creative-reflective contributions

**Fig. 1:**
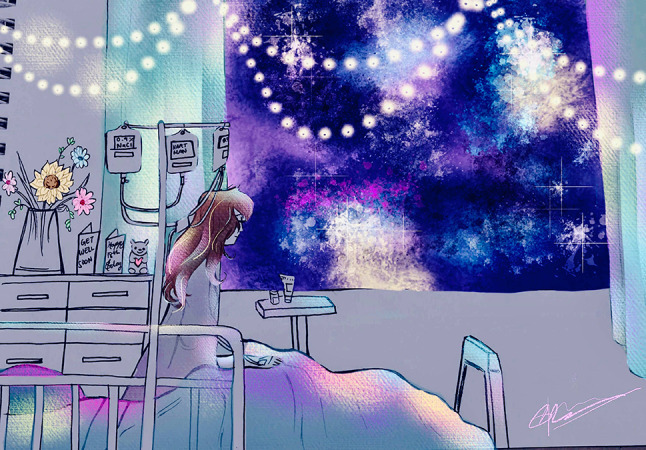
*Escaping Illness with Imagination* by Dilshaayee Prabaharan (3rd year medical student 2019). Dil captures the resilience of a young woman celebrating her 18th birthday in hospital by curating an imaginative gallery through her hospital window as a portal to escape her health struggles

Students and tutors accepted the challenge to re-engage in creative interests and hobbies, and express what vulnerability in medicine means personally. A few students initially found the prospect of developing a creative-reflective contribution daunting but with the support of their tutor and group stepped out of their comfort zone and embraced the challenge. The contributions of students and tutors provided a rich, diverse, and astonishing insight into their humanity and wisdom, which was celebrated in each group. These expressions ranged from poetry, drawing, painting, craftwork, cooking, musical and dramatic performance, to the development of short computer games and digital animations (for an example, see Fig. 1).

[The tutorials] reminded me of the other aspects that make up me … those tucked-away creative parts of me which I realised I had been living without (2020, student 14).

## Impact on clinician-tutors

Tutors participated in regular debriefing sessions and an audio-recorded focus-group discussion. Some tutors were cautious about aspects of the new program:

I was definitely aware of entering an unfamiliar territory. You know, I’ve given a number of different tutorials over the years, but this was something different (Tutor 1).

Several tutors reported higher levels of student respect and engagement than anticipated, and feelings of warmth towards their students:

I’ve been touched by how poignant and thoughtful the students have been in response to the process (Tutor 2).

I feel like it’s required much less selling from me … than I’d anticipated (Tutor 3).

Tutors enjoyed building student confidence in venturing outside their comfort zones. Sharing their own vulnerability in the first tutorial ‘*broke the ice*’ and the explicit tutorial structure and rostering of tutorial tasks fostered a particularly safe, compassionate, and caring space:

They can sense the privilege of this time … with people who are caring, you know, seem to be caring personally for them … you can see them almost steeling themselves towards the time in their practice when they won’t get to do this … when they become a doctor (Tutor 2).

Tutors reported deeper insights into student challenges, hope for the future of the profession, and a reinvigoration of their own clinical and teaching vocations.

## Conclusion

Providing protected time to reflect on the personhood of patients, relationships and challenges in the workplace, including emotions and self-care, is restorative for both students and clinicians. Distinctive aspects of our program include its weekly schedule embedded in specific clinical placements, the semi-structured approach with rostered tasks, prompts to generate discussion, tutors undertaking the same work as students in the spirit of shared leadership, and engagement in creative enquiry. Despite some tutor speculation about whether whole-person care approaches, compassion and self-care can be sustained beyond these supportive spaces, several encounters with former students now working as junior doctors suggest the tutorials have made a positive, enduring impression. We plan to pilot a modified program to clinical teachers.
